# Combining a guided self-help and brief alcohol intervention to improve mental health and reduce substance use among refugee men in Uganda: a cluster-randomized feasibility trial

**DOI:** 10.1017/gmh.2024.103

**Published:** 2024-11-08

**Authors:** M. Claire Greene, Lena S. Andersen, Marx R. Leku, Teresa Au, Josephine Akellot, Nawaraj Upadhaya, Raymond Odokonyero, Ross White, Peter Ventevogel, Claudia Garcia-Moreno, Wietse A. Tol

**Affiliations:** 1 Program on Forced Migration and Health, Heilbrunn Department of Population and Family Health, Columbia University Mailman School of Public Health, New York, NY, USA; 2 Global Health Section, Department of Public Health, University of Copenhagen, Copenhagen, Denmark; 3 HealthRight International, Kampala, Uganda; 4 HealthRight International, New York, NY, USA; 5 Department of Psychiatry, Makerere University, Kampala, Uganda; 6 School of Psychology, Queen’s University Belfast, David Keir Bldg, 18–30 Malone Rd, Belfast BT9 5BN; 7 Public Health Section, Division of Resilience and Solutions, United Nations High Commissioner for Refugees, Geneva, Switzerland; 8 Department of Sexual and Reproductive Health and Research, World Health Organization, Geneva, Switzerland

**Keywords:** alcohol, ASSIST, mental health, self-help plus, refugee, men

## Abstract

Evidence on the effectiveness and implementation of mental health and psychosocial support (MHPSS) interventions for men in humanitarian settings is limited. Moreover, engagement and retention of men in such interventions has been challenging. Adaptations may therefore be required to improve the appropriateness and acceptability of these interventions for men. This study conducted formative research and examined the feasibility of combining an MHPSS intervention, Self-Help Plus, with a brief intervention to reduce harmful alcohol use among refugee men in Uganda. We conducted a cluster randomized feasibility trial comparing the combined alcohol intervention and Self-Help Plus, Self-Help Plus alone and enhanced usual care. Participants were 168 South Sudanese refugee men in Rhino Settlement who reported moderate or high levels of psychological distress. Session attendance was adequate: all sessions had at least 69% of participants present. Participant outcome measures, including symptoms of psychological distress, functional impairment, self-defined problems, depressive symptoms, post-traumatic stress symptoms, overall substance use risk, substance specific risk (alcohol, cannabis, stimulants and sedatives) and well-being, were sensitive to change. A combined approach to addressing mental health and alcohol use appears feasible among men in refugee settings, but further research is needed to examine the effectiveness of combined interventions among men.

## Impact statement

Refugees experience many of the risk factors for mental health problems and alcohol use disorder (AUD), including socioeconomic adversity, exposure to potentially traumatic events and disrupted social networks. Mental health and alcohol use problems often co-occur, yet few of the commonly implemented interventions in humanitarian settings address these conditions concurrently. In this study, we aimed to test the feasibility of delivering a combination of two scalable interventions that have demonstrated the ability to reduce harmful alcohol use and improve mental health, respectively, among men living in refugee settlements in northern Uganda. The first, the ASSIST-linked brief intervention, is a single-session intervention based on motivational interviewing and designed to reduce alcohol use and related problems. The second, Self Help Plus, is a five-session, group-based intervention that uses pre-recorded audio exercises and an illustrated self-help book to promote stress management techniques intended to increase psychological flexibility and reduce distress. Through a qualitative, formative phase, we adapted these interventions and developed a model for integrating them within the study context. We then conducted a cluster randomized feasibility trial with 168 South Sudanese refugee men with moderate to high levels of distress to compare the combined approach, Self-Help Plus only or enhanced usual care. We observed adequate levels of session attendance and sensitivity to change in most mental health and substance use outcomes. These findings indicate that it is feasible to combine evidence-based interventions to address co-occurring mental health and alcohol use problems among men in a refugee settlement context. Combined approaches have the potential to be cost-effective and efficient options to address related and commonly co-occurring problems, such as mental health problems and AUDs. Further research to estimate the cost-effectiveness and other implementation outcomes is needed to determine the value and impact of combined interventions in humanitarian settings.

## Introduction

Forced displacement due to armed conflict poses heightened risks for adverse mental health outcomes (Siriwardhana et al., 2014). Men in humanitarian settings seem particularly vulnerable to substance use disorders including alcohol use disorders (AUDs) (Ezard et al., 2011). Among South Sudanese refugees in northern Uganda, an AUD prevalence rate of roughly 1 in 6 (or 17%) has been documented, with men exhibiting higher levels of AUD (Roberts et al., 2011). A range of factors contribute to AUD including exposure to potentially traumatic events (Roberts et al., 2011), coexisting psychological distress (Lien et al., 2016), feelings of hopelessness (Roberts et al., 2011), unemployment (Roberts et al., 2011; Lien et al., 2016), difficult living conditions (Ezard et al., 2011), availability of alcohol (Roberts et al., 2011) and the feeling of social connectedness associated with ‘drinking together’ (Ssebunnya et al., 2020).

Alcohol use and AUDs pose a significant health challenge in humanitarian settings (Chiumento et al., 2020; Greene et al., 2021). Not only do they compromise the health and well-being of the individual, but they also compromise the community due to the risk of interpersonal violence (Horn, 2010; Ezard et al., 2011; Roberts et al., 2011; Rubenstein et al., 2020; Logie et al., 2022) and child neglect (Hanna, 2017). The urgent need to address common mental disorders has been well established, and mental health and psychosocial support (MHPSS) interventions have been integrated and tested in humanitarian programming (Bangpan et al., 2019).

There is a notable lack of evidence regarding effective MHPSS interventions specific to men, including interventions addressing psychological distress and AUDs (Greene et al., 2019, 2021, 2023). For example, research on a global scale has established that men are less likely to seek care, including mental healthcare, compared to women (Galdas et al., 2005). A previous study conducted among South Sudanese refugees in northern Uganda reported difficulties engaging and retaining men in MHPSS interventions (Tol et al., 2018). A systematic review of MHPSS intervention trials conducted in humanitarian settings found that only 4 out of 29 studies focused on men, mainly targeting former soldiers (Purgato et al., 2018). Men are underrepresented in MHPSS intervention research in humanitarian settings, and interventions may require adaptations to improve their acceptability and relevance for men (Riley, 2023).

AUDs and mental disorders are often comorbid and their symptoms are highly related, yet most interventions often target specific mental health conditions (Kaysen et al., 2023) despite evidence that AUDS and depression are best treated in combination (DeVido and Weiss, 2012). There is a particular need for interventions that address both AUDs and the underlying psychological distress reported by many men in humanitarian settings. This could either be in the form of a transdiagnostic intervention that targets characteristics that underlay both conditions or in the form of a combined intervention that brings together evidence-based psychological interventions for both conditions. Either approach could improve outcomes for both conditions, while also improving the efficiency of dissemination of one rather than multiple intervention protocols in resource-limited settings (Lydecker et al., 2010; Barlow et al., 2017).

The World Health Organization (WHO) developed the ASSIST brief intervention (ASSIST-BI) to address mild to moderate AUDs in low resource settings (Humeniuk et al., 2008). This intervention, based on motivational interviewing, has been implemented and tested in many parts of the world including in humanitarian settings with good outcomes. However, it only targets AUD use and does not address co-morbid psychological distress. Similarly, the WHO developed the Self-Help Plus (SH+) stress management intervention to address psychological distress in people impacted by adversity (Epping-Jordan et al., 2016). This intervention has demonstrated good outcomes in several settings (Purgato et al., 2019). This includes a cluster randomized controlled trial (cRCT) conducted among South Sudanese refugee women in northern Uganda (Tol et al., 2020). However, the formative work leading up to this trial, which included men and women, revealed critical considerations for interventions with male refugees. First, it became clear that it was imperative in this context to address alcohol use alongside psychological distress. Second, further adaptations were needed to the study design to retain men in the intervention. During piloting, only 38% of men attended four to five sessions compared to a 76% attendance rate among women participants (Tol et al., 2018).

To address this need for a scalable intervention addressing both alcohol use and psychological distress for men in humanitarian settings, we proposed combining and testing two existing evidence-based interventions: ASSIST-BI and SH+. Combining two existing interventions builds upon promising psychological techniques and saves time and resources by utilizing existing manuals and materials. We hypothesize that combining these interventions will also facilitate engagement and promote the mental health benefits of SH+ by motivating reductions in harmful alcohol use through the ASSIST-BI. These interventions are freely accessible and can be adapted for use in specific populations. The objectives of the current study were therefore to conduct 1) formative work to adapt the combined ASSIST-BI and SH+ intervention model and 2) a feasibility cRCT to test relevance, safety, acceptability and feasibility of intervention and research protocols of SH+ combined with ASSIST-BI.

## Methods

### Study setting

South Sudan has experienced a long history of violent conflict and displacement stemming from the struggle for independence from Sudan. The country has endured two civil wars spanning decades until 2011 when autonomy was granted to South Sudan. However, independence has been followed by internal conflicts that have resulted in human rights abuses, widespread loss of life, massive displacement and one of the largest and most underfunded humanitarian crises in the world. Although a peace agreement was signed in 2018, challenges remaining in maintaining peace and displacement, food insecurity and ongoing instability continue to impact the region (Tankink et al., 2023). More than 2 million South Sudanese refugees have fled the country due to the crisis, over 880,000 of whom are currently hosted within Uganda (United Nations High Commissioner for Refugees, 2023).

Rhino Refugee Settlement is located in the Arua district of northern Uganda and is one of the largest refugee settlements in the country. It was established in 1980 during the civil war in what is now South Sudan. Imvepi Refugee Settlement is situated north of Rhino Settlement. It was established in 2016 in response to overcrowding in nearby refugee settlements. Both settlements accommodate displaced populations from diverse ethnic groups, almost all from South Sudan (United Nations High Commissioner for Refugees, 2018). The settlements rely on humanitarian assistance by the government, UN agencies and non-governmental organizations, providing essential services, including healthcare. The settlements face ongoing challenges including limited resources, overcrowding and lack of opportunities for self-reliance through employment and livelihood activities (Adaku et al., 2016).

## The intervention: self-help plus combined with ASSIST-BI

### SH+

The WHO developed SH+ as a potentially scalable and accessible intervention to address psychological distress (Epping-Jordan et al., 2016). SH+ is a group-based, self-help stress management course that utilizes pre-recorded audio and an illustrated self-help book, reducing the need for specialized mental healthcare professionals and allowing for larger groups to participate. The course consists of five weekly 90-min sessions covering skills of grounding, unhooking, acting on your values, being kind and making room. The sessions include individual exercises and small group discussions and are facilitated by a lay counselor. The self-help book covers the key points from the course and is provided to participants to review between sessions. Based on principles of acceptance and commitment therapy (ACT), SH+ aims to increase psychological flexibility and reduce distress and help people live in accordance with personal values (Epping-Jordan et al., 2016).

### ASSIST-BI

The WHO’s ASSIST-BI is an evidence-based approach for addressing substance use (Humeniuk et al., 2012). It involves the administration of a screening tool, the Alcohol, Smoking and Substance Involvement Screening Test (ASSIST), to assess substance use risk. This is followed by a single brief one-on-one intervention, based on motivational interviewing techniques, wherein personalized feedback is provided on the individual’s substance use risk to help them understand the impact of substance use on different areas of their lives including their health. After the brief intervention, individuals are provided with a take-home workbook with exercises and strategies for reducing or stopping substance use.

### Adaptations

Prior research has indicated that psychological interventions, such as SH+, for men needed to also address alcohol use (Tol et al., 2018). Using a locally adapted version of SH+ implemented in a previous trial with South Sudanese refugee women, further adaptations were made to improve the relevance for men both with and without risky alcohol use (i.e., tailoring examples, using alternative awareness exercises available in the appendix of the SH+ manual). For men with moderate-risk alcohol use defined by the ASSIST measure, we introduced the ASSIST-BI intervention to accompany SH+. These adaptations were designed through an iterative process of community consultations, cognitive interviewing, facilitator training and uncontrolled pilot/mock sessions with exit interviews, described in more detail below.

#### Translation, cognitive interviewing and facilitator training

SH+ had already been translated into Juba Arabic and adapted for South Sudanese people using culturally relevant examples and illustrations in the women’s trial (Tol et al., 2018, 2020).The ASSIST-BI manual for providers and the intervention materials were translated into Juba Arabic by a bilingual speaker with extensive translation experience and back-translated by another experienced bilingual speaker to check for inconsistencies. Using Van Ommeren’s translation monitoring approach (van Ommeren et al., 1999), these adapted materials were then reviewed by a South Sudanese mental health specialist to ensure concept translation and further adapted.

The original version of the ASSIST-BI includes strategies for reducing or stopping drinking that are provided in a workbook to be completed at home. This workbook requires a level of literacy to learn about strategies to motivate behavior change that is inaccessible to those who cannot read and write. We decided to extend the length of the ASSIST-BI session to include a review of these strategies within the one-on-one session with the facilitator to better equip participants to change their drinking behavior and remove the literacy requirement. This extended the session by approximately 10 min. We created a handout (to replace the handbook) consisting of visuals with written prompts that summarized the information and strategies that we shared with the participants during the extended ASSIST-BI session. We created a flipbook of the intervention content for the facilitators to use when administering the intervention to improve fidelity and provide content-specific visuals to increase participant engagement and understanding during the session.

The revised and translated SH+ and ASSIST-BI materials underwent cognitive interviewing through focus groups with community members and MHPSS providers to ensure understanding, relevance and comprehensibility of the materials. Further adaptations were made prior to training facilitators in the intervention materials. A social worker from the women’s trial, experienced with administering the SH+ intervention, trained three additional facilitators in SH+. A clinical psychologist trained all four facilitators in the ASSIST-BI intervention. Training took place over a 3-day period, in person for SH+ and remotely for ASSIST-BI. During training, facilitators identified further changes that were needed and these were incorporated into the materials.

#### Uncontrolled pilot study

The purpose of the uncontrolled pilot was to test the implementation of the adapted SH+ and ASSIST-BI materials and modify them, as needed, prior to the feasibility trial. Participants were 50 South Sudanese refugee men who spoke Juba Arabic and/or English, were 18+ years of age and had at least moderate distress as indicated by a minimum score of five on the K6 (Kessler et al., 2010). We did not restrict the sample to men reporting moderate or high-risk alcohol use given that the ASSIST-BI intervention is intended to support a range of interventions for individuals at all risk levels from providing feedback and reinforcement to people who report low-risk or no alcohol use to providing feedback, brief intervention and, in some cases, referral to services for people with moderate or high alcohol use risk. Exclusion criteria consisted of high risk of suicide as assessed by the MINI 5.0 suicide module (Sheehan et al., 1998), observable signs of severe mental illness (e.g., psychosis) that could impede participation or high risk substance use (e.g., dependence requiring specialized treatment). Participants excluded for these reasons were referred to the Psychiatric Clinical Officer (PCO) for further assessment. Participants were recruited and screened by a research assistant using the WHO-ASSIST and K6 and using a range of recruitment methods to identify the most acceptable approaches (e.g., door-to-door, recruitment from places where men gather). To evaluate implementation and research procedures, we recruited two cohorts of men (*n* = 30 and *n* = 20), and each cohort participated in mock sessions.

Recruitment during the uncontrolled pilot was feasible with door-to-door recruitment being the favored recruitment strategy reported by the research assistants. The implementation of the intervention sessions was also successful, and no negative events (such as disruptions or attending sessions under the influence of alcohol) were reported by the facilitators. During the exit interviews following the mock sessions, all participants reported that the session content was applicable, helpful and acceptable including the visual depictions, which were created by a local artist. All participants described a motivation to decrease or abstain from drinking alcohol following the ASSIST-BI session, and some specifically mentioned that they had not previously known the negative health impacts of alcohol and drugs. Participants did not provide any recommendations or suggestions for improvements to the intervention, so further adaptations were not deemed necessary.

## Feasibility cluster randomized controlled trial participants and procedures

We conducted a cRCT in six villages with 176 South Sudanese refugee men in Rhino Refugee Settlement in northwestern Uganda. A cluster design was chosen to minimize chances of contamination by participants sharing intervention content and materials within villages. The MildMay Uganda Research Ethics Committee (MUREC) approved the study. All participants provided informed consent.

The eligibility criteria for the feasibility trial were the same as for the uncontrolled pilot study as specified above. Men were primarily recruited door-to-door as this was the recruitment strategy we found to be most feasible and favored during the uncontrolled piloting phase of the current study. Recruitment had to be expanded from six villages within Rhino Refugee Settlement to eight villages in Rhino and Imvepi Refugee Settlements to ensure there were enough eligible men to screen. Two villages (*n* = 60) were randomized to receive SH+ only. Two villages (*n* = 60) were randomized to receive enhanced usual care (EUC). Four villages were randomized to receive ASSIST-BI and SH+ (*n* = 56).

### Enhanced usual care

Participants who were enrolled within the two villages assigned to EUC completed the baseline assessment and were then offered a health visit by a research assistant. The health session consisted of a 30-min session in the participant’s home on the effects of psychological distress, simple strategies to manage overthinking, feedback on their ASSIST score with simple advice to stop or reduce alcohol use, services available in and nearby Rhino Refugee Settlement and how to access them. Participants in the experimental study conditions also received the health session. Participants in EUC completed a follow-up assessment administered by a research assistant at 7-week post-enrollment.

### SH+

Participants enrolled within the two villages assigned to SH+ only completed the baseline assessment, received the health visit and were invited to attend five weekly SH+ group sessions. These participants completed a 7-week post-baseline (1-week post intervention) assessment administered by a research assistant.

### ASSIST-BI and SH+

Participants enrolled within the four villages assigned to ASSIST-BI and SH+ completed the baseline assessment and received the health visit and were invited to attend the individual ASSIST-BI session followed by five weekly SH+ group sessions. These participants also completed a 7-week post-baseline (1-week post intervention) assessment administered by a research assistant.

ASSIST-BI sessions were delivered one-to-one at the location preferred by the participant, which in most cases was at their homes. Group sessions were delivered by a pair of lay facilitators. The facilitators were supervised remotely by a Uganda-based clinical psychologist. A social worker was on-site to provide immediate support if urgent matters arose. Qualitative interviews (*n* = 31) were also conducted post-treatment with participants, both those who completed and those who did not complete the treatment, with family members of the participants, and with the facilitators and the clinical psychologist who provided remote supervision to the facilitators. Table 1 displays the specific interventions that were provided as a function of study arm and participant levels of psychological distress and alcohol risk level at baseline.

**Table 1.** Overview of interventions provided in the three proposed study arms

Study arms	Participant psychological distress and substance use at baseline		
	Elevated psychological distress and low substance-related risk	Elevated psychological distress and moderate substance-related risk	Low psychological distress and moderate substance-related risk
EUC	EUC	EUC	EUC (not eligible)
SH+	EUC and SH+	EUC and SH+	EUC (not eligible)
ASSIST–BI and SH+	EUC, SH+, ASSIST–BI general health information	EUC, SH+ ASSIST–BI	ASSIST–BI (not eligible)

## Outcomes and measures

The primary outcomes of the feasibility trial included feasibility of recruitment (i.e., percentage of men screened who are eligible and who meet criteria for low-, moderate- or high-risk drinking), intervention engagement and attrition, contamination and psychometric performance of participant outcome measures. A program monitoring form was used to track recruitment, enrollment and session attendance. Contamination across sites was assessed using the locally developed contamination form, which was used in the previous SH+ trial among South Sudanese women. Participant outcomes measures included the alcohol and other drug use (WHO-ASSIST), psychological distress (K6), functional impairment (WHODAS), self-defined problems and well-being (PSYCHLOPS), depressive symptoms (PHQ-9), post-traumatic stress symptoms (PCL-6), subjective well-being (WHO-5) and psychological flexibility (AAQ-II). All the secondary outcome measures had been translated from the prior trial with women (Tol et al., 2020). A visual analogue scale consisting of drinking glasses with increasing amounts of water in them was used alongside all measures using Likert scale responses. The ASSIST measure was translated using Van Ommeren’s translation monitoring process through the use of the translation monitoring form, which captures the content, semantic, technical, criterion and conceptual equivalence of each item between the English and Juba Arabic versions (van Ommeren et al., 1999). In addition to translating each question, local names and types of substances (e.g., khat) were included under relevant substance categories. Substances that were not relevant or known in this context (e.g., heroin) were removed. The adapted ASSIST measure was reviewed by a South Sudanese mental health specialist and in two focus groups, one each with community members and with local MHPSS providers to ensure the relevance, comprehensibility and acceptability of the questions and the response options. All measures were administered by a trained bilingual research assistant.

### WHO-ASSIST

The World Health Organization Alcohol, Smoking and Substance Involvement Screening (WHO-ASSIST) test is a validated eight-item measure designed to assess individuals’ involvement and risk level with various substances including alcohol, tobacco and other substances (Humeniuk et al., 2008). Based on the responses to the questions on substance use patterns, the measure provides a continuous score of the level of risk (low, moderate and high) associated with use of alcohol, tobacco and other substances. The ASSIST has been used extensively in alcohol use research globally and among South Sudanese refugees specifically (Roberts et al., 2011).

### K6

The Kessler (K6) scale is a six-item measure designed to assess non-specific psychological distress (Kessler et al., 2010). It asks about the frequency of symptoms such as feeling nervous, hopeless, restless, depressed, worthless and overwhelmed in the past 30 days. Each question is rated on a five-point Likert scale from 0 (none of the time) to 5 (all of the time), and the scores are summed to an overall score of psychological distress, with higher scores indicating greater distress. The K6 demonstrated acceptable psychometric properties among South Sudanese refugee women in the previous SH+ trial (Tol et al., 2020).

### WHODAS

The World Health Organization Disability Assessment Schedule (WHODAS) is a 36-item measure of functional ability and disability (World Health Organization, 2010). It assesses an individual’s ability to perform and participate in various activities, including when living with a health condition, across the six domains of cognition, mobility, self-care, getting along with others, life activities (household, leisure and work-related) and participation in society. Responses are rated on a five-point Likert scale ranging from 1 (none) to 5 (extreme or cannot do). Each domain score can range from 4 to 20, and an overall score is provided by summing the domain-specific scores, with higher scores indicating a greater degree of disability (Ustun et al., 2010).

### PSYCHLOPS

The Psychological Outcome Profiles (PSYCHLOPS) is a three-item questionnaire of the psychological well-being and functioning of individuals (Ashworth et al., 2004). The assessment consists of three open-ended questions that allow the individual to describe and rate their own problems and psychological concerns, the extent to which these problems affect their daily life and their overall well-being. The responses for questions 2 (how much are these difficulties or problems affecting your daily life?) and 3 (how would you rate your overall well-being?) are rated from 0 (not at all) to 10 (extremely), providing a quantitative measure of the individual’s subjective experience. The PSYCHLOPS has been used in clinical settings and in research, including among forcibly displaced populations, to help in the evaluation of treatment effectiveness.

### PHQ-9

The Patient Health Questionnaire (PHQ-9) is a valid and reliable nine-item measure of depressive symptoms (Kroenke et al., 2001). It is based on the Diagnostic and Statistical Manual of Mental Disorders (DSM-5) and consists of questions that ask about sadness, anhedonia, changes in appetite, sleep patterns, feelings of worthlessness or guilt, difficulties concentrating and thoughts of self-harm in the past 14 days. Responses are rated on a four-point Likert scale from 0 (not at all) to 3 (nearly every day) and are summed to provide a total score ranging from 0 to 27, with higher scores indicating more severe depression.

### PCL-6

The Posttraumatic Stress Disorder Checklist (PCL-6) is a six-item measure of the presence and severity of post-traumatic stress disorder (PTSD) symptoms which has shown good psychometric properties (Lang and Stein, 2005). It is based on the DSM-5 and consists of questions that cover the core symptoms of PTSD including intrusive thoughts or memories, avoidance of reminders, negative changes in affect or cognition and heightened arousal or reactivity. Responses are rated on a five-point Likert scale from 0 (not at all) to 4 (extremely) with the total score ranging from 0 to 24, with higher scores indicating greater severity.

### WHO-5

The WHO-5 Well-Being Index is a valid five-item measure of subjective well-being and emotional functioning (Topp et al., 2015). The measure asks about positive mood, vitality and general well-being in the past 2 weeks. Responses are rated on a six-point Likert scale ranging from 0 (not present) to 5 (constantly present) with the total score ranging from 0 to 30, with higher scores indicating greater well-being.

### AAQ-II

The Acceptance and Action Questionnaire (AAQ-II) is a seven-item measure of psychological flexibility (Bond et al., 2011). The AAQ-II is based on the principles of ACT, which emphasizes the importance of psychological flexibility, that is, the capacity to accept difficult thoughts, feelings and bodily sensations without trying to control or avoid them while committing to actions that align with one’s values and goals even in the presence of discomfort or distress. Responses are rated on a seven-point Likert scale ranging from 1 (never true) to 7 (always true) with a total score ranging from 0 to 49, with higher scores indicating higher levels of psychological flexibility.

## Analysis

We examined the distribution of alcohol and other substance use risk levels and psychological distress among all men who were screened to evaluate the feasibility of recruitment. Using this information, we estimated the proportion of screened men who were eligible to participate, which served as an indicator of recruitment feasibility and efficiency. To describe intervention attendance, we calculated the number of participants attending each session in the overall sample and by study condition. We explored the feasibility of allocation and randomization procedures by comparing the distribution of demographic and clinical characteristics, including demographic characteristics, substance use patterns and psychological distress, across the study conditions using chi-square and ANOVA analyses. We explored whether contamination or misallocation of study participants occurred using the program monitoring form. Sensitivity to change of study outcomes over time was estimated using mixed-effects models that included random intercepts for village and participant. For each of the study outcomes, we calculated and reported the instrument’s internal consistency using Cronbach’s alpha.

## Results

### Characteristics of study sample at baseline

One hundred eighty-six men from nine villages were screened for eligibility. The original six villages selected for the study were expanded to nine villages in order to identify enough adult men for screening to reach our target sample size. Among those screened, 176 were eligible, enrolled and completed a baseline assessment. Of the 10 excluded men, 6 declined to participate and 4 did not meet inclusion criteria. The four ineligible participants were excluded due to possible serious mental illness (*n* = 2) and suicide risk (*n* = 2) and were referred to the local PCO for further assessment. Eleven of the 176 enrolled participants reported low risk for all substance use. The remaining were classified as being at moderate or high risk of substance use for one or more of the substances assessed in this study (Table 2). We observed low risk levels for most substance categories including cannabis (89.9%), cocaine (98.3%), other stimulants (76.4%), inhalants (98.3%), sedatives (96.7%) or other substances (99.3%). Most participants were categorized as moderate risk for tobacco use problems (64.6%) followed by low risk (31.5%) or high risk (3.9%). Most participants similarly were classified as moderate risk for AUD (58.0%), followed by low risk (33.5%) or high risk (8.5%).

**Table 2.** Distribution of ASSIST total and substance specific scores among screened participants

Construct	Mean (SD)	Low risk *N* (%)	Moderate risk *N* (%)	High risk *N* (%)
Total substance involvement score	40.54 (25.12)	–	–	–
Tobacco	12.12 (9.83)	57 (31.49)	117 (64.64)	7 (3.87)
Alcohol	14.13 (10.01)	63 (33.51)	109 (57.98)	16 (8.51)
Cannabis	1.63 (5.29)	161 (89.94)	18 (10.06)	0 (0.00)
Cocaine	0.25 (1.93)	176 (98.32)	3 (1.68)	0 (0.00)
Stimulants	3.82 (7.78)	139 (76.37)	38 (20.88)	5 (2.75)
Inhalants	0.18 (1.56)	176 (98.32)	3 (1.68)	0 (0.00)
Sedatives	0.39 (2.39)	174 (96.67)	6 (3.33)	0 (0.00)
Other substances	0.06 (0.49)	140 (99.29)	1 (0.71)	0 (0.00)

All 176 enrolled participants were allocated to one of the following study conditions based on their village of residence: EUC (*n* = 60), SH+ only (*n* = 60) and SH+ and ASSIST-BI (*n* = 56). Enrolled participants had a mean age of 36.0 years (SD = 13.15). Most were Kakwa ethnicity (59.1%), had more than a primary school education (52.3%), were currently married (76.1%) and were employed (73.6%). All of these demographic characteristics differed significantly across study conditions at baseline (*p* < 0.05; see Table 3).

**Table 3.** Characteristics of sample by study arm

	EUC *K* = 3 villages *N* = 60 participants	SH+ only *K* = 2 villages *N* = 60 participants	SH+ and ASSIST-BI *K* = 4 villages *N* = 56 participants	*F*/*X* ^2^ (*p)*
Age, *M* (SD)	31.57 (11.69)	38.52 (12.28)	35.79 (13.87)	4.25 (0.016)
Ethnicity, *n* (%)				46.41 (<.001)
Dinka	0 (0.00)	0 (0.00)	9 (16.07)	
Kakwa	41 (68.33)	38 (67.86)	22 (39.29)	
Pojulu	6 (10.00)	17 (30.36)	8 (14.29)	
Other	13 (21.67)	1 (1.79)	17 (30.36)	
Marital status, *n* (%)				43.06 (<.001)
Married	46 (76.67)	47 (83.93)	37 (66.07)	
Never married	9 (15.00)	5 (8.93)	14 (25.00)	
Separated/divorced/widowed	5 (8.34)	4 (7.15)	5 (8.93)	
Education				112.06 (<.001)
No schooling	1 (1.67)	2 (3.57)	5 (8.93)	
Primary school	33 (55.00)	25 (44.64)	15 (26.79)	
Junior school	4 (6.67)	10 (17.86)	8 (14.29)	
Senior school/senior high school	18 (30.00)	11 (19.64)	23 (41.07)	
College/diploma/university	4 (6.67)	8 (14.29)	5 (8.93)	
Employment, *n* (%)				47.30 (<.001)
Farmer	37 (61.67)	18 (33.33)	20 (35.71)	
Other employment (mechanic, paid work, pastor, student, house worker, etc.)	7 (11.67)	8 (14.81)	10 (17.86)	
Self–employed	5 (8.33)	18 (33.33)	9 (16.07)	
Unemployed	11 (18.33)	10 (18.52)	17 (30.36)	
Psychological distress (K–6), *M* (SD)	13.02 (4.02)	13.18 (3.89)	14.29 (4.21)	1.87 (0.157)
Functioning (WHODAS), *M* (SD)	27.40 (7.70)	30.61 (9.14)	28.71 (8.57)	2.10 (0.125)
Self–defined problems (PSYCHLOPS), *M* (SD)	16.50 (2.49)	16.50 (2.86)	16.73 (3.26)	0.17 (0.841)
Depressive symptoms (PHQ–9), *M* (SD)	11.52 (4.89)	13.46 (5.07)	13.18 (5.16)	2.50 (0.085)
PTSD symptoms (PCL–6), *M* (SD)	18.48 (5.69)	18.59 (6.17)	18.43 (6.24)	0.07 (0.936)
Subjective well–being (WHO–5), *M* (SD)	7.75 (4.48)	7.73 (5.21)	7.71 (5.71)	0.02 (0.982)
Psychological flexibility (AAQ–II), *M* (SD)	33.32 (10.27)	33.68 (11.27)	33.30 (10.25)	0.00 (0.997)
Total substance involvement (ASSIST TSI), *M* (SD)	42.60 (24.10)	39.64 (24.47)	24.48 (22.55)	1.74 (0.179)
Tobacco use (ASSIST), *M* (SD)	13.93 (9.57)	11.71 (9.85)	9.52 (9.02)	3.15 (0.045)
Alcohol use (ASSIST), *M* (SD)	14.24 (9.21)	13.98 (9.51)	12.82 (10.11)	0.31 (0.733)
Cannabis use (ASSIST), *M* (SD)	2.12 (6.17)	1.15 (3.99)	1.88 (5.86)	0.61 (0.543)
Cocaine use (ASSIST), *M* (SD)	0.00	0.39 (1.59)	0.41 (3.07)	0.75 (0.475)
Stimulants use (ASSIST), *M* (SD)	4.57 (7.65)	2.34 (5.70)	2.70 (6.30)	1.95 (0.146)
Inhalants use (ASSIST), *M* (SD)	0.15 (1.17)	0.43 (2.52)	0.00	0.94 (0.394)
Sedatives use (ASSIST), *M* (SD)	0.12 (0.65)	1.07 (4.16)	0.07 (0.38)	2.79 (0.064)
Other substance use (ASSIST), *M* (SD)	0.00	0.00	0.12 (0.78)	0.63 (0.536)

**Table 4.** Sensitivity to change and internal consistency of participant-level outcome measures

	Baseline *M* (SD) (*n* = 176)	Endline *M* (SD) (*n* = 159)	Mean change (95% CI)	Cohen’s *d*	ICC (within participant)	ICC (within village)	Cronbach’s alpha
K–6	13.43 (4.02)	12.07 (5.55)	−1.13 (−2.13, −0.13)	0.28	0.206	0.053	0.714
WHODAS	28.89 (8.50)	24.03 (8.40)	−4.66 (−6.51, −2.83)	0.58	0.171	0.007	0.818
PSYCHLOPS	16.55 (2.84)	13.73 (5.16)	−2.38 (−3.25, −1.51)	0.69	0.041	0.041	0.724
PHQ–9	12.69 (5.07)	8.65 (5.79)	−3.57 (−4.82, −2.33)	0.75	0.002	0.002	0.744
PCL–6	18.57 (5.96)	15.38 (5.84)	−3.00 (−4.26, −1.74)	0.54	0.162	0.000	0.853
WHO–5	7.78 (5.10)	10.48 (6.59)	2.36 (1.08, 3.64)	−0.46	0.054	0.010	0.767
AAQ–II	33.36 (10.47)	29.17 (11.02)	−3.55 (−5.88, −1.22)	0.39	0.115	0.010	0.864
ASSIST TSI	38.88 (23.63)	30.30 (22.10)	−9.29 (−12.79, −5.79)	0.37	0.610	0.015	0.869
Tobacco	11.73 (9.55)	10.86 (9.69)	−1.28 (−2.92, 0.37)	0.09	0.522	0.005	0.662
Alcohol	13.54 (9.58)	10.25 (10.07)	−3.09 (−4.72, −1.46)	0.34	0.543	0.033	0.403
Cannabis	1.68 (5.37)	0.52 (2.66)	−1.23 (−2.09, −0.38)	0.27	0.366	0.000	0.888
Cocaine	0.26 (1.97)	0.27 (2.37)	−0.13 (−0.26, 0.01)	0.00	0.913	0.000	0.788
Stimulants	3.22 (6.60)	1.91 (5.10)	−2.08 (−3.19, −0.97)	0.22	0.319	0.008	0.766
Inhalants	0.19 (1.59)	0.00	−0.10 (−0.25, 0.06)	0.17	0.812	0.002	0.690
Sedatives	0.41 (2.43)	0.09 (1.19)	−0.26 (−0.48, −0.05)	0.16	0.827	0.000	0.894
Other substances	0.06 (0.50)	0.00	−0.06 (−0.16, 0.04)	0.17	0.000	0.000	0.554

Study outcome measures including psychological distress, functional impairment, self-defined problems, depressive symptoms, post-traumatic stress symptoms, well-being, psychological flexibility and substance use risk did not differ significantly across study conditions at baseline with one exception. At baseline, tobacco risk was higher in the EUC condition relative to the SH+ and ASSIST-BI conditions (*F* = 3.15, *p* = 0.045).

### Intervention attendance and study retention

**Figure 1.**
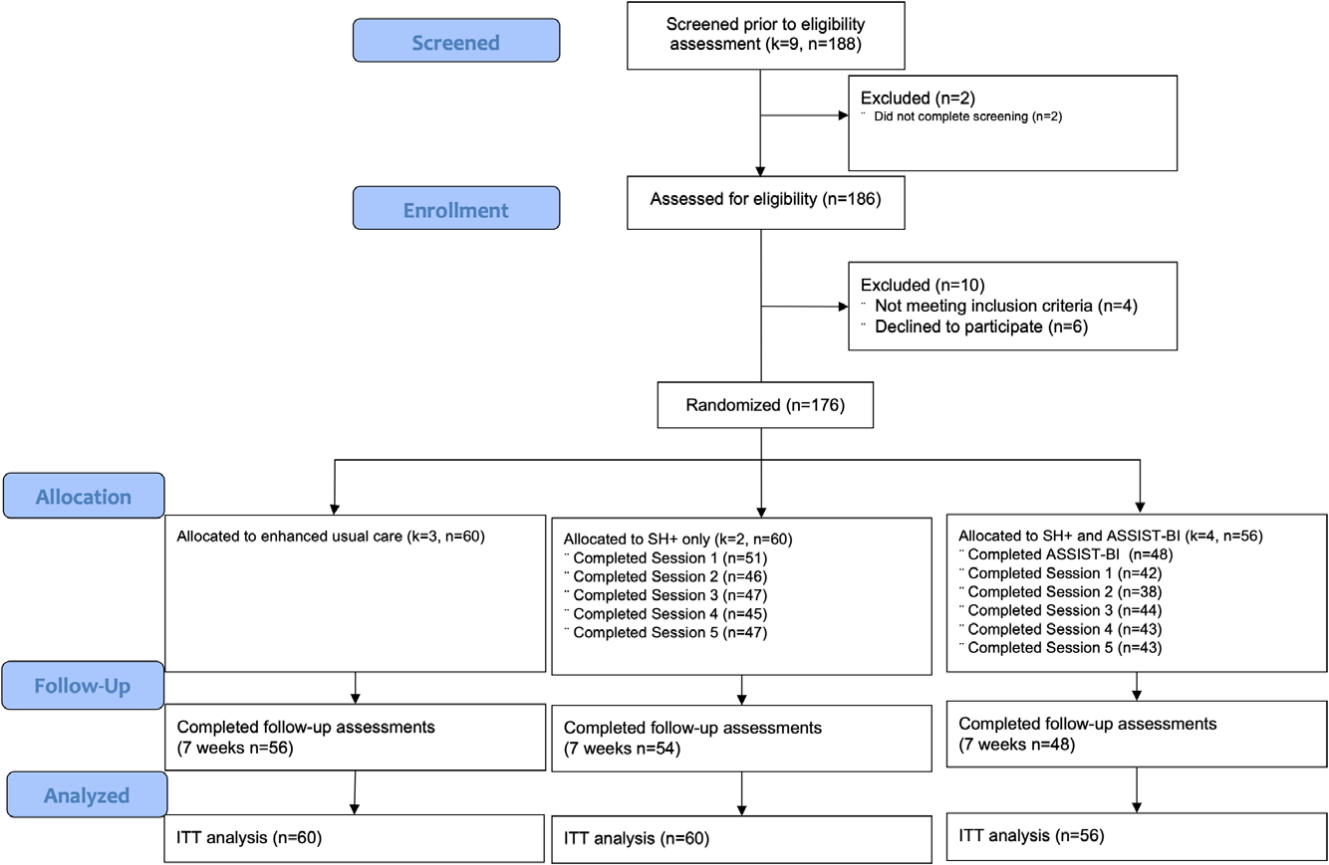
Study flow diagram (*k* = villages, *n* = participants).

As shown in Figure 1, attendance and study retention were high across study conditions. Ninety percent of participants completed the follow-up assessment at 7 weeks post-enrollment (ranging from 85.7% of SH+ and ASSIST-BI participants to 93.3% of EUC participants). In each session, between 75.0% and85.0% of participants were present in the SH+ only group condition and 67.9% and 78.6% in the SH+ and ASSIST-BI group plus one-on-one condition. Feedback reports provided by the facilitators after intervention sessions indicated that participants were engaged, no disruptions occurred, and the recordings and materials were well understood by participants. We did not find any evidence of contamination between study arms, and no adverse events were reported.

### Sensitivity to change in mental health symptoms and substance use outcomes

In the full sample, we observed significant changes in the following participant outcomes that suggest good sensitivity to change (Table 4): symptoms of psychological distress (mean change = −1.13; 95% CI: −2.13, −0.13), functional impairment (mean change = −4.66; 95% CI: −6.51, −2.83), self-defined problems (mean change = −2.38; 95% CI: −3.25, −1.51), depressive symptoms (mean change = −3.57; 95% CI: −4.82, −2.33), post-traumatic stress symptoms (mean change = −3.00; 95% CI: −4.26, −1.74), psychological inflexibility (mean change = −3.55; 95% CI: −5.88, −1.22) and well-being (mean change = 2.36; 95% CI: 1.08, 3.64). Effect sizes were small for psychological distress (*d* = 0.28), well-being (*d* = −0.46) and psychological flexibility (*d* = 0.39). We observed medium effect sizes for functional impairment (*d* = 0.58), self-defined problems (*d* = 0.69), depressive symptoms (*d* = 0.75) and post-traumatic stress symptoms (*d* = 0.54).

Smaller changes were observed for substance use risk. There was a significant change in overall substance use risk level (mean change = −9.29; 95% CI: −12.79, −5.79; *d* = 0.37), alcohol risk (mean change = −3.09; 95% CI: −4.72, −1.46; *d* = 0.34), cannabis risk (mean change = −1.23; 95% CI: −2.09, −0.38; *d* = 0.27) and stimulant risk (mean change = −2.08; 95% CI: −3.19, −0.97; *d* = 0.22) in the overall sample. A smaller, yet statistically significant change was also observed for sedative risk (mean change = −0.26; 95% CI: −0.48, −0.05; *d* = 0.16). We did not observe a sensitivity to change in the risk of other substance use problems including tobacco (mean change = −1.28; 95% CI: −2.92, 0.37; *d* = 0.09), cocaine (mean change = −0.13; 95% CI: −0.26, 0.01; *d* = 0.00), inhalants (mean change = −0.10; 95% CI: −0.25, 0.06; *d* = 0.17) or other substances (mean change = −0.06; 95% CI: −0.16, 0.04; *d* = 0.17). Changes by study condition are reported in Supplementary Table 1.

The intraclass correlation coefficient for all study outcomes was generally higher within participant (range: 0.00–0.91) relative to within village (range: 0.00–0.05).

## Discussion

This study explored the feasibility of combining an adapted self-help intervention to reduce psychological distress with a brief intervention to reduce alcohol and other substance use-related problems. The adaptation and combination of these interventions was done to increase the relevance of these interventions for refugee men in Uganda. We observed high uptake of the intervention as evidenced by the high levels of attendance and low attrition, which supports the utility of this combined approach. These findings highlight the feasibility of using the ASSIST-BI to address harmful substance use in refugee settings, which complements a low-intensity psychological intervention such as SH+. If proven effective, the combination of SH+ and ASSIST could form a useful first-line intervention approach for male refugees with psychological distress and low- to moderate-level substance use risk. This integrated intervention model complements other ongoing research efforts aiming to integrate a higher-resource and higher-intensity alcohol intervention within a scalable problem-solving intervention in Rhino Refugee Settlement, Uganda (Fuhr et al., 2021). These complementary efforts may help to build a stepped care model for psychological distress and co-occurring alcohol and other substance use problems in refugee settings. Future research could test this stepped care approach including screening and referral pathways from the lower to the higher intensity intervention.

This study also highlights important findings related to common substance use patterns among refugee men in Uganda. The most commonly used substances were alcohol and tobacco, which is similar to findings from previous needs assessments conducted in other refugee settlements in Uganda (United Nations Office on Drugs and Crime, 2018). Stimulants (particularly khat) were also commonly used. Several other studies have documented the burden of khat use in refugee and non-refugee samples, particularly in east Africa (Alem et al., 1999; Odenwald et al., 2005; Widmann et al., 2014; Adorjan et al., 2017; Mihretu et al., 2017; Widmann et al., 2017; Ongeri et al., 2019). Most measurement tools and interventions have not been adequately adapted to measure or address khat use. However, the ASSIST-BI has been previously adapted for khat use in Kenya and was found to be acceptable among Somali refugee men. An evaluation of the adapted ASSIST-BI found that the intervention is associated with significant reductions in the amount and frequency of khat use (Widmann et al., 2022). In our sample, polysubstance use was common and over half of participants met criteria for moderate or high risk in more than one substance category. While we observed some small, yet significant changes in substance use risk levels in our overall sample, further research examining the appropriateness of this type of brief intervention for people with polysubstance use is needed.

This study also examined whether the cRCT design is feasible within this study context. We found that the recruitment strategy, particularly door-to-door recruitment, efficiently identified eligible adult men with moderate psychological distress. Most of these men also reported moderate alcohol or other substance use risk for at least one substance. However, during the study we expanded our catchment areas from six to nine villages because we were not able to find enough adult men. Men were highly mobile and often leaving the settlement for work and were therefore not accessible for identification and screening for the study. Flexibility in recruitment timelines, approaches and intervention delivery modalities is required for future fully-powered trials or other studies in similar populations and settings. We also found that the demographic characteristics of study participants differed significantly across study arms, which may reflect the clustering of similar individuals within villages and the heterogeneity of the population composition between villages. Other study designs, such as an individually randomized or stratified cluster randomized trial, must be considered in future research to reduce the risk of confounding and selection bias in a fully-powered randomized trial.

This feasibility trial has some limitations. First, this study was not designed to evaluate the effectiveness of the combined interventions and several adaptations were made to the interventions during the formative phase of this study. For these reasons, we cannot determine whether observed improvements in study outcomes can be attributable to the combined interventions. These findings, however, have helped elucidate important considerations that can inform a future fully-powered randomized controlled trial. Additionally, some of the participant outcome measures have not yet been validated within the male study population and context although all performed well among female refugees. These outcomes were also only measured at two timepoints, limiting our ability to explore when and how changes occurred. Future research on interventions should incorporate mechanism of change tracking for complex interventions to understand how the change is happening (e.g., whether the changes in psychological symptoms precede the reduction in alcohol use). Lastly, this study enrolled men with high levels of psychological distress and a range of alcohol use risk levels. Therefore, we are not able to determine the feasibility of this combined approach or the ASSIST-BI alone for men with low levels of psychological distress. This was done to enable us to explore the feasibility of combining an alcohol intervention with an intervention aimed at alleviating psychological distress to overcome alcohol-related barriers to engagement and participation that were observed in research (Tol et al., 2018).

Despite these limitations, this study provides preliminary evidence of uptake and relevance of this integrated intervention approach for refugee men in Uganda. It also has identified important considerations to improve an evaluation design that can inform a future fully-powered randomized controlled trial.

## Supporting information

Greene et al. supplementary material Greene et al. supplementary material

## Data Availability

The data that support the findings of this study are available from the senior author upon reasonable request.

## Supplementary material

The supplementary material for this article can be found at http://doi.org/10.1017/gmh.2024.103.
