# Combining a guided self-help and brief alcohol intervention to improve mental health and reduce substance use among refugee men in Uganda: a cluster-randomized feasibility trial – CORRIGENDUM

**DOI:** 10.1017/gmh.2024.145

**Published:** 2024-12-16

**Authors:** M. Claire Greene, Lena S. Andersen, Marx R. Leku, Teresa Au, Josephine Akellot, Nawaraj Upadhaya, Raymond Odokonyero, Ross White, Peter Ventevogel, Claudia Garcia-Moreno, Wietse A. Tol

**Keywords:** alcohol, ASSIST, mental health, self-help plus, refugee, men

Upon publication of this article the affiliation for author Ross White was incorrectly listed as Institute of Population Health, University of Liverpool. The correct affiliation should be School of Psychology, Queen’s University Belfast, David Keir Bldg, 18-30 Malone Rd, Belfast BT9 5BN

The article has been updated
