# Supplementary material for: Combining a guided self-help and brief alcohol intervention to improve mental health and reduce substance use among refugee men in Uganda: a cluster-randomized feasibility trial
Source: Glob Ment Health (Camb). 2024 Nov 8;11:e106. doi: 10.1017/gmh.2024.103 (PMC11704368; doi:10.1017/gmh.2024.103)
Supplement: Greene et al. supplementary material [file S2054425124001031sup001.docx]

Supplemental Table 1. Sensitivity to change of participants outcome measures by study arm

|  | Enhanced Usual Care, Mean Change (95 % CI) | SH+ Only,  Mean Change (95 % CI) | SH+ and ASSIST-BI,  Mean Change (95 % CI) |
| --- | --- | --- | --- |
| K-6 | 1.07 (-0.36, 2.51) | -3.65 (-5.47, -1.83) | -1.37 (-2.91, 0.18) |
| WHODAS | -1.57 (-4.44, 1.29) | -7.02 (-10.86, -3.17) | -6.24 (-8.67, -3.82) |
| PSYCHLOPS | -0.56 (-1.77, 0.66) | -4.00 (-5.56, -2.45) | -3.19 (-4.88, -1.50) |
| PHQ-9 | -0.38 (-2.18, 1.42) | -6.46 (-8.70, -4.21) | -4.95 (-7.03, -2.87) |
| PCL-6 | -1.11 (-2.96, 0.74) | -5.08 (-7.40, -2.75) | -3.65 (-5.98, -1.33) |
| WHO-5 | 0.28 (-1.46, 2.02) | 5.46 (3.10, 7.83) | 2.15 (-0.23, 4.53) |
| AAQ-II | 0.37 (-2.98, 3.72) | -10.51 (-15.07, -5.95) | -2.16 (-5.62, 1.30) |
| ASSIST TSI | -8.32 (-14.16, -2.48) | -13.72 (-20.31, -7.13) | -6.86 (-12.25, -1.47) |
| Tobacco | -1.21 (-3.59, 1.18) | -4.53 (-7.37, -1.69) | 1.71 (-1.44, 4.86) |
| Alcohol | -2.15 (-4.64, 0.33) | -4.38 (-7.50, -1.26) | -2.97 (-5.82, -0.12) |
| Cannabis | -1.60 (-2.96, -0.25) | -0.69 (-1.78, 0.40) | -1.45 (-3.42, 0.53) |
| Cocaine | *No cocaine use reported* | -0.37 (-0.88, 0.15) | 0.00 (0.00, 0.00) |
| Stimulants | -2.90 (-4.93, -0.86) | -0.41 (-1.54, 0.71) | -2.58 (-4.69, -0.47) |
| Inhalants | -0.15 (-0.47, 0.16) | 0.00 (0.00, 0.00) | *No sedative use reported* |
| Sedatives | -0.11 (-0.29, 0.06) | -0.57 (-1.22, 0.08) | -0.07 (-0.20, 0.05) |
| Other substances | *No other substance use reported* | 0.00 (0.00, 0.00) | -0.12 (-0.39, 0.15) |
