# Supplementary material for: Combining a guided self-help and brief alcohol intervention to improve mental health and reduce substance use among refugee men in Uganda: a cluster-randomized feasibility trial
Source: Glob Ment Health (Camb). 2024 Nov 8;11:e106. doi: 10.1017/gmh.2024.103 (PMC11704368; doi:10.1017/gmh.2024.103)
Supplement: Peer review history

# Editor report: Recommendation: Combining a guided self-help and brief alcohol intervention to improve mental health and reduce substance use among refugee men in Uganda: a cluster-randomized feasibility trial — R0/PR2

**DOI:** 10.1017/gmh.2024.103.pr2

**Affiliations:** University of KwaZulu-Natal, South Africa

The article requires major revisions especially as outlined by the second reviewer.

# Editor report: Decision: Combining a guided self-help and brief alcohol intervention to improve mental health and reduce substance use among refugee men in Uganda: a cluster-randomized feasibility trial — R0/PR3

**DOI:** 10.1017/gmh.2024.103.pr3

**Affiliations:** Johns Hopkins University Bloomberg School of Public Health, United States

No accompanying comment.

# Editor report: Recommendation: Combining a guided self-help and brief alcohol intervention to improve mental health and reduce substance use among refugee men in Uganda: a cluster-randomized feasibility trial — R1/PR5

**DOI:** 10.1017/gmh.2024.103.pr5

**Affiliations:** University of KwaZulu-Natal, South Africa

Please attend to the additional comments of reviewer 2.

# Editor report: Decision: Combining a guided self-help and brief alcohol intervention to improve mental health and reduce substance use among refugee men in Uganda: a cluster-randomized feasibility trial — R1/PR6

**DOI:** 10.1017/gmh.2024.103.pr6

**Affiliations:** Johns Hopkins University Bloomberg School of Public Health, United States

No accompanying comment.

# Editor report: Recommendation: Combining a guided self-help and brief alcohol intervention to improve mental health and reduce substance use among refugee men in Uganda: a cluster-randomized feasibility trial — R2/PR8

**DOI:** 10.1017/gmh.2024.103.pr8

**Affiliations:** University of KwaZulu-Natal, South Africa

No accompanying comment.

# Editor report: Decision: Combining a guided self-help and brief alcohol intervention to improve mental health and reduce substance use among refugee men in Uganda: a cluster-randomized feasibility trial — R2/PR9

**DOI:** 10.1017/gmh.2024.103.pr9

**Affiliations:** Johns Hopkins University Bloomberg School of Public Health, United States

No accompanying comment.
